# Development and Testing of SPIDER-NET: An Interactive Tool for Brain Connectogram Visualization, Sub-Network Exploration and Graph Metrics Quantification

**DOI:** 10.3389/fnins.2022.818385

**Published:** 2022-03-17

**Authors:** Davide Coluzzi, Alice Pirastru, Laura Pelizzari, Monia Cabinio, Maria Marcella Laganà, Giuseppe Baselli, Francesca Baglio

**Affiliations:** ^1^Dipartimento di Elettronica, Informazione e Bioingegneria, Politecnico di Milano, Milan, Italy; ^2^IRCCS Fondazione Don Carlo Gnocchi Onlus, Milan, Italy

**Keywords:** MRI, brain networks, connectograms, brain connectivity, graph analysis, stroke

## Abstract

Brain connectomics consists in the modeling of human brain as networks, mathematically represented as numerical connectivity matrices. However, this representation may result in difficult interpretation of the data. To overcome this limitation, graphical representation by connectograms is currently used via open-source tools, which, however, lack user-friendly interfaces and options to explore specific sub-networks. In this context, we developed SPIDER-NET (Software Package Ideal for Deriving Enhanced Representations of brain NETworks), an easy-to-use, flexible, and interactive tool for connectograms generation and sub-network exploration. This study aims to present SPIDER-NET and to test its potential impact on pilot cases. As a working example, structural connectivity (SC) was investigated with SPIDER-NET in a group of 17 healthy controls (HCs) and in two subjects with stroke injury (Case 1 and Case 2, both with a focal lesion affecting part of the right frontal lobe, insular cortex and subcortical structures). 165 parcels were determined from individual structural magnetic resonance imaging data by using the Destrieux atlas, and defined as nodes. SC matrices were derived with Diffusion Tensor Imaging tractography. SC matrices of HCs were averaged to obtain a single group matrix. SC matrices were then used as input for SPIDER-NET. First, SPIDER-NET was used to derive the connectogram of the right hemisphere of Case 1 and Case 2. Then, a sub-network of interest (i.e., including gray matter regions affected by the stroke lesions) was interactively selected and the associated connectograms were derived for Case 1, Case 2 and HCs. Finally, graph-based metrics were derived for whole-brain SC matrices of Case 1, Case 2 and HCs. The software resulted effective in representing the expected (dis) connectivity pattern in the hemisphere affected by the stroke lesion in Cases 1 and 2. Furthermore, SPIDER-NET allowed to test an *a priori* hypothesis by interactively extracting a sub-network of interest: Case 1 showed a sub-network connectivity pattern different from Case 2, reflecting the different clinical severity. Global and local graph-based metrics derived with SPIDER-NET were different between cases with stroke injury and HCs. The tool proved to be accessible, intuitive, and interactive in brain connectivity investigation and provided both qualitative and quantitative evidence.

## Introduction

In the last decades, the emergence of -omics disciplines led to the development of flexible and comprehensive methods to effortlessly analyze big data sets. Graph theory is a suitable means for leveraging big data and for modeling complex real-world systems, characterized by specific architecture and topology. This mathematical approach has been effectively applied in several scientific fields. One of the most impressive and popular application is the so called “human connectome” (Bullmore and Sporns, 2009), namely modeling the human brain as a network on many different scales (Rubinov and Sporns, 2010), aiming to connect its structure to function and behavior. Similar to network genomics, which models the influence of genes in a larger biomolecular system, brain connectomics reconfigures the study of brain structure and function by mapping the whole brain in terms of neural units and their connections (Sporns et al., 2005). Indeed, brain regions are strongly connected through neuroanatomical white matter (WM) pathways, intuitively determining a complex system. In parallel to structural connectivity (SC), synchronous and asynchronous activity of specific brain regions results in related complex cognitive functions, which can be investigated in terms of functional connectivity (FC). Exploring SC and FC patterns can provide insight of brain function both in physiological and pathological conditions.

A network is a mathematical representation of a complex system that is defined by a collection of nodes (vertices) and links (edges), describing any kind of relationship between pairs of nodes, at different scales. Networks can be easily represented as n-by-n association matrices, where n is the number of nodes composing the network, while each element e_ij_ represents the link connecting the nodes *i* and *j*. The elements of the matrix can be either binary (i.e., describing the presence/absence of links between pairs of nodes) or weighted (i.e., describing the strength of the links between pairs of nodes). In the framework of the human connectome, association matrices are brain connectivity matrices. According to the technique or imaging modality employed to extract the connectivity data, nodes and edges of a brain network can represent different concepts. When constructing brain connectivity matrices from magnetic resonance imaging (MRI) dataset, nodes usually represent gray matter parcels, defined according to well-known atlases (Tzourio-Mazoyer et al., 2002; Desikan et al., 2006; Smith et al., 2009; Yeo et al., 2011). Brain atlases segment the brain into sets of voxels (i.e., parcels), based either on anatomical or functional criteria. Similarly, the edges of a brain network, describing a relationship between nodes, can depict either SC or FC features. SC refers to anatomical associations between neural elements or brain regions, while FC represents the magnitude of temporal correlations between the signal produced by pairs of brain regions. SC and FC can be quantified with various indices, depending on the imaging modality which is used to investigate the connectivity pattern. For instance, the number of streamlines derived with deterministic WM tractography can be used as weights in an MRI-derived SC matrix, while correlation between blood oxygenation level-dependent (BOLD) time series can be used to define edges of MRI-derived FC matrices. SC and FC generally mirror an undirected relationship between brain regions (i.e., non-causal), resulting in a symmetric connectivity matrix [i.e., (Nx(N-1))/2 pairwise connections between N nodes].

Although brain connectivity matrices can exhaustively and quantitatively outline the human connectome, this representation does not always provide an intuitive and direct visualization of the connectivity pattern. Brain connectivity matrices are generally too large to be visually interpreted, thus important information might remain concealed. For this reason, conceiving new methods for the visualization of connectivity data is needed to aid the interpretation of brain connectivity measures. This is important especially for explorative analyses, with the aim of identifying characteristic patterns that may allow to distinguish the pathological condition from the physiological one, or to assess changes after a pharmacological treatment or rehabilitation.

Connectograms are graphical representations that meet these needs, bridging the gap between quantitative connectivity analyses and intuitive visualization. Connectograms are circular graphs in which all the nodes of a networks are represented along the perimeter of the circle, while the edges of the network are shown as arcs connecting pairs of nodes. This layout was previously used in other fields (e.g., genomics) and it was introduced for brain connectivity mapping by Irimia et al. (2012b) about 10 years ago. Connectograms can be produced using Circos (Krzywinski et al., 2009), which is a powerful software package designed for visualizing data, for exploring relationships between objects and for creating publication-quality illustrations. It is extremely flexible, and it can be used in several diverse fields. However, Circos has no interface, and it has to be run by command-lines. This approach does not create any problem to brain connectivity researchers who are UNIX users, but it may be uncomfortable to researchers who do not have any programming experience. A user-friendly interface that could be used by people having knowledge and interest in brain connectivity but not in computer science may broaden the accessibility to connectograms.

A complete whole-brain network can be made of thousands of links, and it is well-known that this large-scale network is associated with high-level cognitive functions. However, the brain is composed of several interacting lower-scale sub-networks, which are characterized by distinct patterns of brain activation, identifying specific domains of behavior and cognition (Bassett and Sporns, 2017). Therefore, extracting sub-networks is common practice in explorative studies of brain connectivity, both in physiological and pathological conditions (Zalesky et al., 2010; Bassett and Sporns, 2017; Berron et al., 2020; Isernia et al., 2021). Indeed, focusing on sub-networks can lead to easier data interpretation driven by the addressed physiological and/or pathological problem. Sub-network should be analyzed both qualitatively, by a reduced connectogram, and quantitively by local and global subgraph indexes. Nevertheless, software that are currently available for connectivity pattern visualization (e.g., Circos, BrainNet Viewer, Xia et al., 2013) does not allow for the interactive selection of some specific nodes within a whole-brain network, as the direct upload of the sub-network of interest is generally required. Beside outlining the brain connectome through an association matrix and finding an intuitive way to effectively represent it, graph-based network properties can be calculated to depict a complete picture of the architecture of the whole network and of each sub-network of interest. Indices such as node degree, small-worldness, modularity, clustering, and central hubs, can add meaningful information about the network topology. These are valuable pieces of information in a brain connectivity analysis as many studies revealed changes of organizational and topological properties in a number of brain disorders, such as Alzheimer’s Disease (Daianu et al., 2013), mild cognitive impairment (Baggio et al., 2014), Parkinson’s Disease (Göttlich et al., 2013), epilepsy (Ji et al., 2017), autism (Barttfeld et al., 2012) and borderline intellectual functioning (Blasi et al., 2020). Despite the large evidence produced in the last years, gold-standard methodologies are not established yet, and connectivity alterations in neurological and neurodegenerative diseases are an open issue, so far. Therefore, further investigations on human brain networks are warranted. Accessible tools for easily assessing the topology and architecture of brain networks would provide larger amount of evidence that may promote deeper knowledge of brain disorders and of the effect of treatments (e.g., disease modifying therapies, rehabilitation) on brain networks.

In this framework, we developed SPIDER-NET (Software Package Ideal for Deriving Enhanced Representations of brain NETworks), a software package that provides a very flexible and user-friendly tool for the selection of partial connectograms, their visualization, and their quantification. The SPIDER-NET Graphical User Interface (GUI) intuitively allows rapid network exploration and interactive real-time sub-network definition. Figures for connectivity studies are automatically generated, based on the user selections. Furthermore, the toolbox provides additional features to apply matrix thresholding, to easily and automatically compute topological network indices and to interactively define visualization preferences. The aims of this study were: (1) presenting SPIDER-NET, and (2) testing the potential benefits of using SPIDER-NET in clinical research case studies. Specifically, the following aspects were tested: (2a) providing an effective representation of brain connectivity patterns, (2b) interactively extracting sub-networks to test *a priori* hypotheses and (2c) deriving whole-brain quantitative connectivity metrics mirroring local and global topological properties.

## Materials and Methods

### SPIDER-NET Overview

**FIGURE 1:**
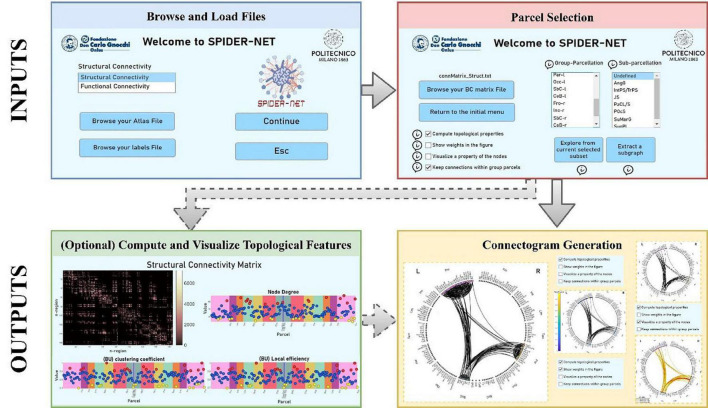
Flowchart for SPIDER-NET usage. First, the Atlas and Label input files are browsed and loaded (blue box). Then, the Connectivity Matrix file is loaded and the selection of the sub-network of interest is performed (red box). Optionally (dashed lines), it is possible to compute and visualize topological properties of the selected brain network (green box). Finally, the connectogram is generated according to the selection made and to the chosen visualization settings (yellow box).

SPIDER-NET was developed in Matlab but is delivered as a standalone software (.exe in Windows, .app in macOS, .sh in UNIX). The tool allows flexible and effective representation of brain networks through connectograms. It enables the exploration of network architecture and topology and, optionally, the extraction of topological properties describing the network architecture and nodes properties. A schematic flowchart is shown in Figure 1.

### SPIDER-NET Inputs

SPIDER-NET requires 3 input files, which are an Atlas file, a Label file and a Connectivity Matrix file.

1) The Atlas file is an XSL/XSLX Excel worksheet that provides information on the atlas the user adopts to define the network nodes. The list of the Atlas parcels is reported in a column of the worksheet. All the parcels listed in the Atlas file are reported as nodes in the connectogram generated by SPIDER-NET. The sorting of the parcels in the Atlas file determines the positions of the parcels in the connectogram obtained with SPIDER-NET (i.e., the first parcel is represented on the top of the circle). A short legend has to be associated to each parcel in the Atlas file. The legend is shown in the interface to help interactive node selection. Reporting optional parcel grouping (i.e., Group Parcellation) is also allowed in the Atlas file (e.g., brain lobes, resting state networks). In additional columns (i.e., Attribute), the Atlas file can enclose additional optional attributes associated with each parcel (e.g., functional attribute). Both Group Parcellation and Attributes can be used to rapidly select entire groups of parcels (i.e., parcels sharing Group Parcellation tag or Attribute tag) for sub-network extraction. Several Atlas files, for the most used structural and functional atlases, are provided as templates together with the software. Moreover, it is possible to customize or create new Atlas files according to the user’s preferences and aims, by simply composing new worksheets.
2) The Label file (ASCII text file, .txt) is a list of the parcel names. The order of parcels in this file must strictly repeat the order of rows and columns in the Connectivity Matrix, which is the third input for SPIDER-NET. Therefore, the order of the parcels in the Label file is tied to the Connectivity Matrix generation. The design choice of repeating the same list of parcels in both the Atlas file and in the Label file permits great flexibility for connectogram generation, as the same Atlas file can be used with many different Label files (and Connectivity matrices). Indeed, all the parcels being equal, the order of parcels can vary across Label files (according to the associated Connectivity matrices), while the sorting of the parcels in the circular representation remains the same if the same Atlas file is used for generating the connectograms.
3) The Connectivity matrix file is an ASCII text file, containing the matrix of association weights with row and column order matching with the Label file. Any measure of SC or FC derived with MRI is applicable. The input connectivity matrix must be square (NxN) and symmetric. Conventionally, the main diagonal must be set to zero.

Once the inputs have been uploaded (Figure 1, blue box), the selection of either single parcels, entire group-parcels or attributes defined in the “Atlas” file, is enabled in the SPIDER-NET GUI (Figure 1, red box).

### Parcel Selection and Connectogram Generation

Two complementary logics are offered by SPIDER-NET in the interactive definition of a partial network out of the global one represented by the input Connectivity matrix (Figure 1, red box): “Explore from current selected subset” (Option 1) and “Extract a subgraph” (Option 2).

When the interactive definition of the addressed sub-network is completed on the GUI, with either Option 1 or Option 2, SPIDER-NET generates the partial connectogram (Figure 1, yellow box). The figure of the connectogram was designed by reengineering and extending the circular graph package developed for Matlab (GitHub. Retrieved July 28, 2021).^1^ This library allows to draw nodes along a circumference, and their connections, whose shape is defined by the Poincaré hyperbolic disk (Gao et al., 2020).

The connectogram figure generated by SPIDER-NET is automatically saved in a folder created at run-time for each execution. However, interactive changes to the resulting connectogram are also possible within the software figure-management GUI. Specifically, changes improving readability of too crowded diagrams are available: (i) single nodes can be selected to hide/show the respective labels; (ii) connections related to specific nodes can be temporarily removed from the connectogram. The modified figures can be saved in addition to the original one.

#### Option 1—Explore From Current Selected Subset

One or more “seed” parcels are defined. Next, the set of target parcels is defined, which can be either all the parcels of the brain, just some other parcels or a specific group of parcels (i.e., defined by Group Parcellation or any additional Attribute defined in the Atlas file). It is worth noting that in the set of target parcels is also possible to include the parcels already selected as “seed.” Only the edges between each seed-target pair are represented in the resulting connectogram. SPIDER-NET deals with non-directional graphs, so “seed” and “target” are fully conventional names. This option can be useful for the analysis of alterations due to focal lesions or for qualitative pilot quality control of the processing pipeline, to check for major errors in the connectivity matrix generation. For instance, this option can be used in a preliminary quality check of structural connectivity data verifying the presence of connections between a chosen seed and all the other parcels which are linked to it by existing WM tracts, basing upon anatomical knowledge. This can be particularly valuable given the complex image acquisition and upstream processing.

#### Option 2—Extract a Subgraph

This option is based on the selection of a single subset of nodes (i.e., brain parcels). The user can select either parcels one by one, or entire groups of parcels, defined according to Group Parcellation or any additional Attribute reported in the Atlas file. Therefore, after the selection, a subgraph is defined with respect to the original Connectivity matrix, and connections between all the possible pairs of the selected parcels are shown in the resulting connectogram. This kind of exploration is useful when specific cerebral circuits are addressed or deeper verification of single well-known connections in the quality control of the pipeline are requested.

#### Additional Features

Although SPIDER-NET has been designed as an easy-to-use software GUI for simple connectogram generation, additional optional features were developed and can be set simultaneously to parcel selection (Figure 1, red box).

First, if the parcels are grouped according to a higher-level classification (e.g., brain lobes) in the Atlas file, links between pairs of parcels belonging to the same group (e.g., Group Parcellation) can be optionally omitted from the connectogram. Excluding within-group links from the connectogram enables a clearer visualization of long-range connections, especially when many connections are displayed.

Second, SPIDER-NET allows to optionally visualize color-coded properties of the nodes, which can be either local graph-theory based (e.g., node degree) or representing other properties of the parcels (e.g., cortical thickness, parcel volume, classification according to functional circuits). Edge properties (i.e., strength of connection) may also be color-coded.

Furthermore, density-based thresholding is commonly applied to matrices in brain connectivity studies, to either remove spurious connections and/or to binarize a weighted matrix. Although the user can provide SPIDER-NET with an already thresholded connectivity matrix, the software is designed to allow for optional density-based thresholding at run-time. In particular, once the user has selected the desired density, the software iteratively searches for the best threshold to approximate the selected density, starting from zero. The thresholded matrix is then used to draw the connectogram.

### Compute and Visualize Topological Features

Another feature optionally implemented by SPIDER-NET (Figure 1, green box) is the computation of graph-based topological indices for a quantitative assessment. Local, global, and intermediate structure (i.e., community detection, core-periphery analysis, rich-clubs) analyses are performed for a total number of 20 computed indices, computed basing on Brain Connectivity Toolbox (Rubinov and Sporns, 2010).^2^ Importantly, the computed indices refer to both the original Connectivity Matrix and to the currently selected subset of nodes, thus providing targeted quantification of cerebral circuits. Besides the connectogram, the following quantitative information is graphically represented when graph-based topological indices are optionally computed:

1. Connectivity weights, shown as a color-coded connectivity matrix.
2. Local indexes (i.e., node degree, clustering coefficient, local efficiency for both the binary and weighted case), shown in plots. The horizontal axis reports the parcel names in the same order as around the connectogram, while the vertical axis scales the local index values. The local index value for each parcel is represented with dots. The index value is color-coded to highlight the most and the least influential nodes. Namely, the top 10% parcels and the least 10% ones are highlighted in red and yellow, respectively. These plots can be effective in pinpointing network hubs or, conversely, lesion related drops.
3. Values of the global indices, listed below the plots of local indices.

These graph-based outputs can be interactively explored, selecting specific elements of the connectivity matrix or dots of local indexes to obtain additional information (i.e., weights, corresponding higher-level classification). It is worth remarking that results of the interactive subgraph analyses are always shown on the screen in parallel to results of the analysis of the original complete connectivity matrix. Once the interactive process is fulfilled, whole graph and subgraph results are saved.

### SPIDER-NET Application on Case Studies

#### Participants

The dataset consists of two patients with stroke injury characterized by a right hemisphere lesion with prevalent subcortical expression (males, age 44 and 37 years old, referred to as Case 1 and Case 2, respectively) and 17 healthy control (HCs) subjects (7 males and 10 females; mean age ± *SD*: 52.5 ± 8.3 years). All the subjects were enrolled at IRCCS Fondazione Don Carlo Gnocchi in Milan and signed a written informed consent.

#### Magnetic Resonance Imaging Acquisition and Matrix Construction

All the participants underwent a MRI examination performed on a 1.5 T Siemens Magnetom Avanto scanner equipped with a 12-channels head coil. Both patients with stroke injury were scanned six months after hemorrhagic stroke.

The acquisition protocol included:

1. a high-resolution 3D T1-weighted Magnetization Prepared Rapid Gradient-Echo (MPRAGE) image, (repetition time (TR)/echo time (TE) = 1,900/3.37 ms, Field of View (FoV) = 192 × 256 mm^2^, resolution = 1 × 1 × 1 mm^3^, 176 axial slices);
2. a diffusion-weighted echo planar images (EPI) image along 64 directions (*b*- value 1,500 s/mm^2^, TR/TE 7,800/109 ms, matrix size = 102 × 102 × 46, resolution = 2.5 × 2.5 × 2.5 mm^3^) and 3 b0 images (2 with AP, and 1 with PA encoding direction);
3. a dual-echo turbo spin echo proton density PD/T2-weighted image (TR = 4,540 ms, TE = 28/112 ms, matrix size = 320 × 320 × 60, resolution = 0.75 × 0.75 × 2 mm^3^).

After standard preprocessing, 3D T1-weighted volumes were parcellated, at subject-level, and automatically labeled into 75 cortical parcels for each hemisphere (150 in total) according to the Destrieux atlas (Destrieux et al., 2010) using FreeSurfer (version 6). Seven subcortical regions per hemisphere (thalamus, caudate, putamen, pallidum, nucleus accumbens, amygdala and hippocampus) and the brainstem were also segmented using the FreeSurfer automatic labeling process (Fischl et al., 2002) for a total of 165 parcels.

Diffusion-weighted images were preprocessed using the FMRIB’s Software Library (FSL) tools with a standard pipeline (i.e., correction for susceptibility-induced geometric distortions, for eddy current distortion and head movements) (Pelizzari et al., 2019) and diffusion tensor imaging (DTI) was estimated for each voxel using the FSL DTIFIT toolbox (Andersson et al., 2003; Behrens et al., 2003; Andersson and Sotiropoulos, 2016). Then, DTI-derived whole brain tract was generated. In addition, diffusion weighted data were processed also with Constrained Spherical Deconvolution (CSD) approach. Specifically, StarTrack^3^ was used both to estimate the fiber orientation distribution function and to perform subsequent deterministic whole brain tractography, according to high angular resolution diffusion imaging (HARDI) processing (Dell’Acqua et al., 2010).

Cortical and subcortical parcels, obtained from the 3D T1-weighted images, were registered to the respective diffusion-weighted space using the FSL flirt toolbox (Jenkinson et al., 2002). Then, for each subject, WM tracts connecting each pair of registered parcels were reconstructed with TrackVis software,^4^ basing both on DTI-derived whole brain tract and CSD-derived whole brain tract.

In both patients, the stroke lesions were segmented by an experienced operator on the PD/T2 volumes with Jim software.^5^

DTI-based and CSD-based SC matrices were derived for patients with stroke injury and HCs, by computing the edges as the number of the reconstructed fiber (NF) of each WM tract connecting each pair of the 165 parcels. In order to account for differences in brain volumes, NF was normalized by the sum of the volumes of the pair of respective connected parcels (Blasi et al., 2020). A probabilistic group matrix was computed to represent the HC group as a whole, retaining only the connections shared by at least 53% of the HCs subjects (Blasi et al., 2020). Therefore, three matrices where finally obtained for both DTI and CSD approach: one for each patient with stroke injury and one HC group matrix. The obtained matrices were then normalized by the respective maximum edge value, so that the matrix elements ranged from 0 to 1.

#### Running SPIDER-NET

SPIDER-NET inputs were defined as follows. The Atlas file was constructed based on the Destrieux atlas.

The Label file, matching the connectivity matrices, reported the Destrieux atlas parcel names. The normalized connectivity matrices for Case 1, Case 2 and HCs (Connectivity matrix files) were uploaded one at a time, for each separate analysis.

First, for the two patients with stroke injury, DTI-based connectograms showing the connectivity pattern in the right hemisphere, where the lesions of both subjects are located, were generated to test the ability of SPIDER-NET to show altered connectivity patterns due to a focal lesion.

Second, for each patient with stroke injury, a DTI-based subgraph focused on expected altered circuit was extracted. Specifically, all the parcels overlapping with the stroke lesions were selected as seeds, while all the remaining brain parcels were set as target for this sub-network analysis. The same sub-network was investigated for the two stroke cases and for the HC group to enable a comparison. The following regions of interest were considered as seeds: right precentral gyrus, right long insular gyrus and central insular sulcus, right short insular gyri, right caudate nucleus, right pallidum, right putamen, and right thalamus. This analysis was performed to test the ability of SPIDER-NET to visually explore sub-networks of interest. Furthermore, the same sub-network analysis was performed for connectivity matrices derived with CSD processing, to compare the connectivity results based on DTI and CSD processing techniques.

Third, the main local and global graph-analysis indices describing network topology were extracted both for the weighted and for the binary connectivity DTI-based matrices. The node degree was the main focus relevant to local graph index analysis.

## Results

### Connectogram Visualization of the Connectivity Pattern Altered by Stroke Lesions

**FIGURE 2:**
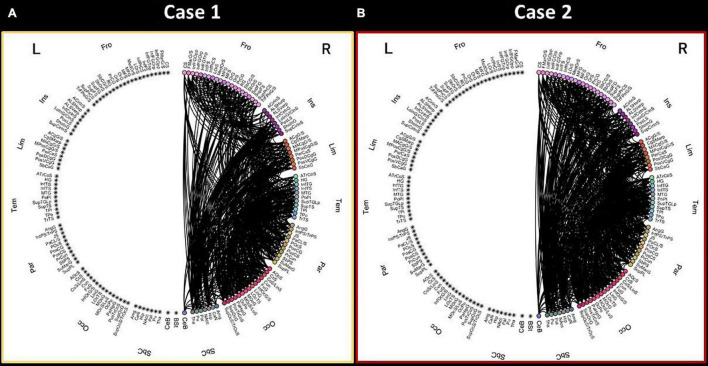
Connectograms showing the connectivity pattern of the right hemisphere for the two stroke patients. The connectogram of Case 1 **(A)** is reported on the left, while the connectogram of Case 2 **(B)** is represented on the right. L-left hemisphere, R-right hemisphere, Fro-frontal, Ins-insular, Tem-temporal, Par-parietal, Occ-occipital, Sbc-subcortical, CeB-cerebellum, Bst-brainstem. Brain parcels are reported with standard labels provided for the Destrieux atlas.

The connectograms showing the DTI-based connectivity pattern of the right hemisphere of the two patients with stroke injury are shown in Figure 2. All the 165 parcels (cortical parcels of Destrieux atlas and subcortical regions) are reported in the circular representation and divided in 8 anatomical lobes (i.e., Group Parcellation defined in the Atlas file) per hemisphere.

Upon visual inspection, the connectivity pattern of the right hemisphere is different in Case 1 compared to Case 2. Specifically, Case 1 shows a less dense connectivity pattern in the right hemisphere, especially in terms of connections among the frontal lobe, insular cortex and subcortical structures.

### Connectograms Visualization for Sub-Network Analysis

**FIGURE 3:**
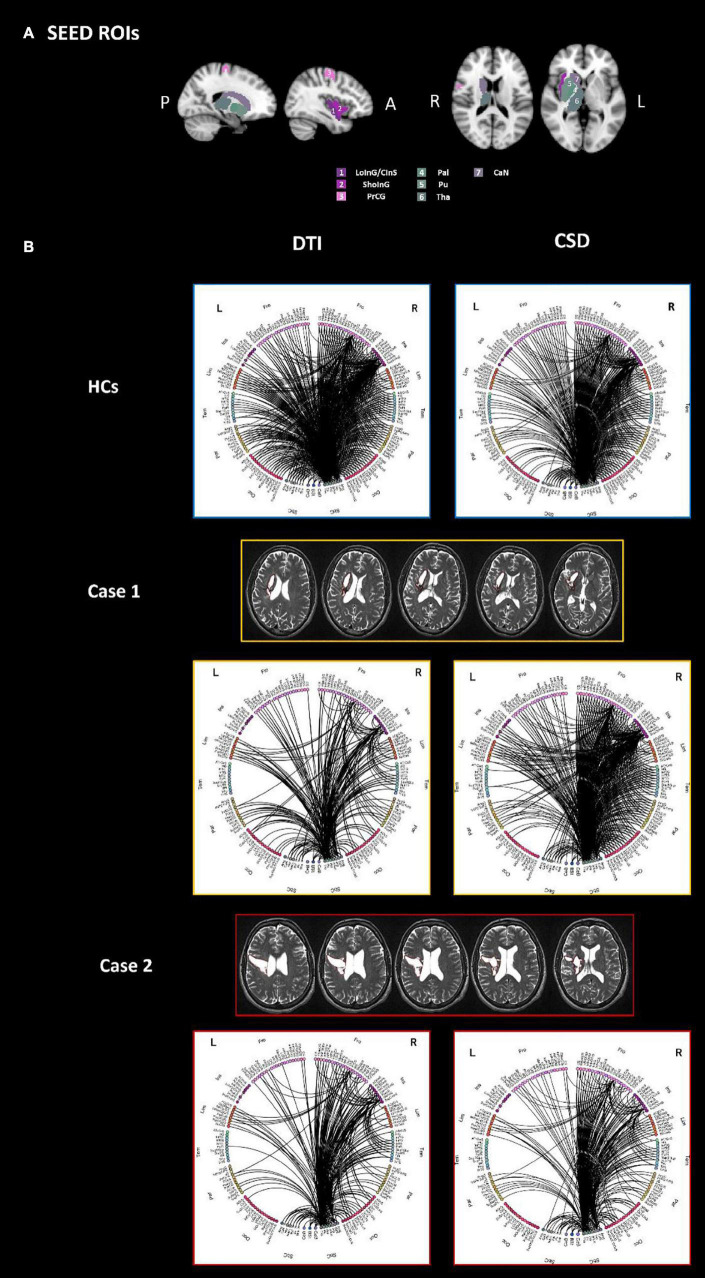
The connectograms **(B)** derived by both DTI (left) and CSD (right) processing, of the sub-network extracted using as seeds all parcels which are overlapped with the stroke lesion of either Case 1 or Case 2, are reported for HCs (top panel), Case 1 (middle panel) and Case 2 (bottom panel). Specifically, seeds were defined as follows: right LoInG/CInS, right ShoInG, right Pal, right Pu, right CaN, right PrCG, right Tha **(A)**. All the other parcels of the brain were considered as target for the connectivity analysis. L-left hemisphere, R-right hemisphere, Fro-frontal, Ins-insular, Tem-temporal, Par-parietal, Occ-occipital, Sbc-subcortical, CeB-cerebellum, Bst-brainstem, PrCG-precentral gyrus, LoInG/CInS-long insular gyrus and central insular sulcus, ShoInG-short insular gyri, Pal-pallidum, Pu-putamen, CaN-caudate nucleus, Tha-thalamus.

The connectograms generated with SPIDER-NET to explore the DTI-based connectivity between gray matter parcels intersecting the lesions of patients with stroke injury (Figure 3A) and the rest of the brain are shown in Figure 3B. The same connectograms obtained with Circos software^6^ are reported in Supplementary Material to allow for comparison.

Upon visual inspection, the sub-network connectivity pattern of both patients with stroke injury looks altered compared to HCs. In addition, it is worth noting that differences can be qualitatively observed both in the right hemisphere (where the stroke lesions are present) and in the contralateral one. Case 1 displays a less dense right hemisphere connectivity pattern compared to Case 2. On the other hand, the sub-network connectogram of Case 2 qualitatively shows less connections in the left frontal-insular area and in the left parietal-occipital lobes compared to Case 1.

#### Diffusion Tensor Imaging-Based and Constrained Spherical Deconvolution-Based Connectivity: Visual Comparison

The connectivity between regions overlapping the lesions of stroke patients and the whole brain was also investigated from matrices derived with CSD processing. The resulting connectograms are shown in Figure 3B.

The sub-network connectograms of both Case 1 and Case 2 looks different when compared to the HC group one, as for the DTI-based connectograms. At visual inspection, the connectograms derived from DTI and CSD processing generally preserve the same connectivity patterns. CSD-based connectograms highlight the difference between Case 1 and Case2 connectivity pattern.

### Local and Global Topological Properties Analysis

**FIGURE 4:**
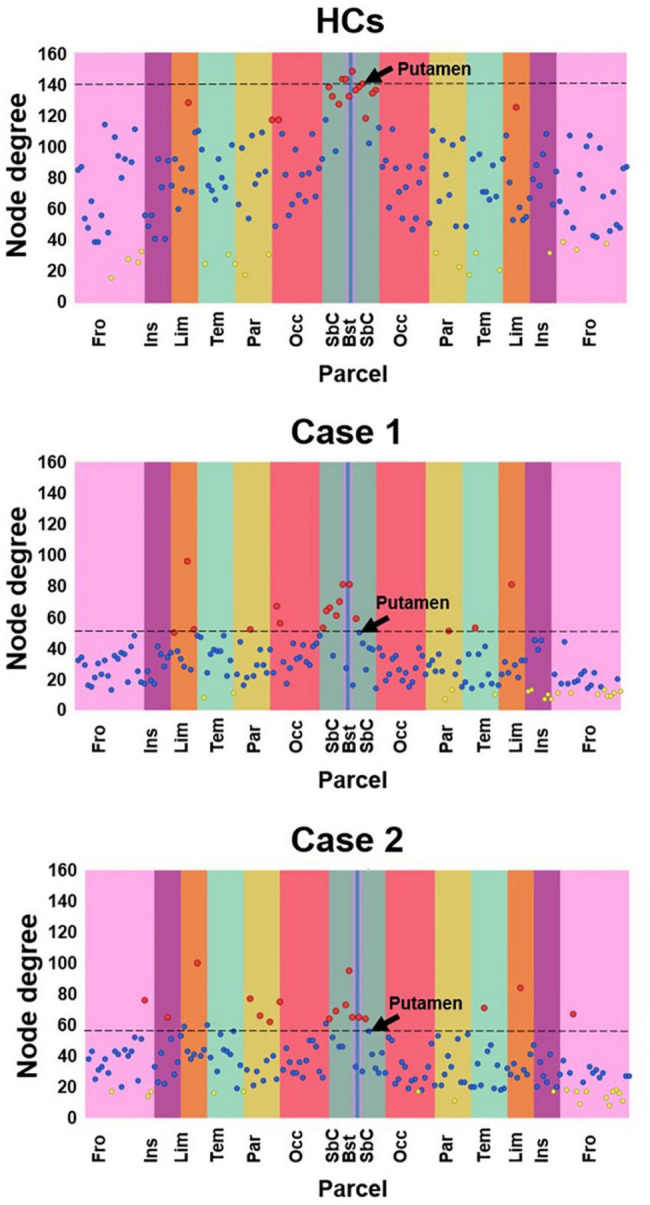
Local node degree computed for HCs, Case 1 and Case 2. The X-axis represent the 165 brain parcels, even if only lobe labels are reported (e.g., Fro). The left hemisphere is represented in the left half of the graph, while the right hemisphere is represented in the right half. Vertical colored stripes represent different lobes (e.g., the frontal lobe is represented in pink). The same lobe in left and right hemispheres is shown with the same color. Each dot in the graph represents the local node degree of a brain parcel. The 17 parcels (10% of 165) exhibiting the highest local node degree are represented as red dots. The 17 parcels (10% of 165) with the lowest local node degree are represented as yellow dots. All the other parcels are represented as blue dots. The SPIDER-NET interactive interface allows to visualize information about each dot, navigating on them. The right Putamen is highlighted by an arrow.

Node degree explorative figures produced by SPIDER-NET for Case 1, Case 2 and HCs are shown in Figure 4. In general, patients with stroke injury presented lower node degrees when compared with HCs. In HCs, regions with the highest node degrees, represented as red dots in Figure 4 (upper panel), were mostly located in the dark-green vertical stripe, representing subcortical regions. Conversely, in the patients with stroke injury, both characterized by a right hemisphere lesion with prevalent subcortical expression, the regions showing the highest local node degree are more distributed across the cortical lobes. In addition, caudate nucleus, pallidum, putamen, and thalamus, which were classified by SPIDER-NET as regions with the highest node degree in HCs (red dots), were not classified as nodes with high node degree in both Case 1 and Case 2. In Figure 4, putamen node degree values are highlighted for HCs, Case 1 and Case 2. Patients with stroke injury presented lower network density (18.65 and 22.32%, respectively, for Case 1 and Case 2) with respect to HC (47.51%), resulting in differences of 28.9 and 25.2%. Global topological binary and weighted indices extracted from the whole-brain network of HC, Case 1 and Case 2 are shown in Table 1.

**TABLE 1:** Global graph-based topological properties of the HC template and the two stroke cases. Reported Delta values were computed as (HC-Case)*100/HC.

Graph-based indexes	HCs	Case 1	HCs-Case1 Delta (%)	Case 2	HCs-Case2 Delta (%)
Average degree	77.915	30.582	60.7%	36.606	53.0%
Average strength (W)	3.048	1.233	59.5%	1.396	54.2%
Clustering coefficient	0.76	0.614	19.2%	0.604	20.5%
Clustering coefficient (W)	0.019	0.017	10.5%	0.015	21.1%
Characteristic path length	1.542	2.092	–35.7%	1.927	–25.0%
Characteristic path length (W)	18.302	35.211	–92.4%	28.544	–56.0%
Global efficiency	0.735	0.549	25.3%	0.587	20.1%
Global efficiency (W)	0.068	0.039	42.6%	0.045	33.8%
Small-worldness	1.591	2.881	–81.1%	2.531	–59.1%
Modularity	0.193	0.38	–96.9%	0.351	–81.9%
Coreness statistic	0.321	0.402	–25.2%	0.367	–14.3%

Both Case 1 and Case 2 presented differences for all the global topological indices when compared to HCs. Percentage differences ranged from 10.5 to 96.9% for Case 1, and from 14.3 to 81.9% for Case 2. Except for clustering coefficient, Case 1 presented with greater percentage difference with HCs when compared to Case 2.

## Discussion

In this study we presented SPIDER-NET, an innovative tool for exploring and visualizing brain connectivity through full and partial connectograms. The tests on two readily interpretable cases with stroke injury proved that this tool is capable of producing meaningful connectograms and of interactively extracting and analyzing focused sub-networks.

As previously mentioned, connectograms were introduced by Irimia et al. (2012a; 2012b) to provide intuitive and clear visualization of neuroconnectivity relationships, alternatively to large numerical matrices which do not allow prompt inference or hypothesis testing about either network properties or pathological damage. Although a variety of tools exploiting connectograms for studying connectomics already exist (Krzywinski et al., 2009; Whitfield-Gabrieli and Nieto-Castanon, 2012; Nieto-Castanon, 2020), so far, none of them allowed both interactive network exploration and the selection of sub-networks, while also providing a user-friendly interface. The interactivity allows for a faster execution of the tool and its use at ease, without the need of recompilation and re-uploading of the files. The user is also guided through the selection of different parameters providing a description for each feature and preventing from possible incidental choices. Therefore, SPIDER-NET could broaden the access to connectivity investigation to any interested user in the neuroscience field (e.g., neuropsychologists, physicians), other than computer scientists.

In the presented application examples, SPIDER-NET allowed to highlight different connectivity patterns between two patients with stroke injury. Interestingly, although both patients were characterized by a right hemisphere stroke lesion with prevalent subcortical expression, the disconnection patterns of the whole right hemisphere looked different between them. The different connectivity pattern of the right hemisphere was highlighted thanks to appropriate subgraph selections, interactively allowed by the tool. As expected, after considering the lesion patterns, right hemisphere connectivity differences qualitatively observed in the connectograms included connections with frontal lobe, insular cortex, and subcortical structures. Of note, although the connectograms presented in the figures were very dense, we chose to maintain the original density of the networks (19.65 and 22.32%, respectively, for Case 1 and 2) before any selection. This was carried out to avoid the possible introduction of thresholding biases, thus reducing the capability to capture the main differences between the connectivity patterns of the two patients, especially considering the low original values of density. Although dense connectograms could result in poor readability, SPIDER-NET solves this issue allowing both interactive exploration of the network and stringent selection as successively performed by analyzing a sub-network of interest.

Indeed, in addition to a first explorative visual investigation of the right hemisphere, SPIDER-NET was used to perform a more focused analysis on a sub-network of interest, interactively testing an *a priori* hypothesis. The comparison of HCs, Case 1, and Case 2 sub-network connectograms generated by SPIDER-NET confirmed the *a priori* hypothesis and provided additional information about the disconnectivity pattern of Case 1 and Case 2, which may be used to improve the understanding of clinical manifestations and to drive personalized treatment. Indeed, both patients presented facio-brachio-crural hemisyndrome, with main brachial expression and severe functional limitation of movements of the left upper limb, especially of the hand. However, this limitation was more severe in Case 1 than in Case 2. This was mirrored by different residual connectivity patterns showed by SPIDER-NET connectograms. The following additional aspects were highlighted thanks to the circular diagrams. First, the pattern of disconnection involved both the right hemisphere, where the stroke lesions were present, and the contralateral one. Second, for both Case 1 and Case 2, the impairment of the cortical areas of interest determined a decrease in both short-range (within lobe) and long-range (between lobes) connections within the hemisphere ipsilateral to the stroke lesion. Third, in both patients with stroke injury the pattern of interhemispheric connectivity was also compromised, probably because subcortical nuclei, which are integration hubs of extrapyramidal systems, were extensively affected by the lesions. Therefore, producing connectograms on focused sub-networks with SPIDER-NET allowed to overcome the difficulty of visualizing the large number of edges that would be present in the whole-brain connectograms.

At a visual inspection, DTI-based and CSD-based sub-network connectograms presented comparable connectivity patterns, highlighting that valuable information is provided by both the processing techniques. Furthermore, CSD processing pipeline yielded to reconstruct denser connectograms, as expected. Indeed, CSD ability to better deal with the problem of the crossing fibers when compared to DTI is well-established (Dell’Acqua et al., 2007). This is in line with differences between DTI and CSD that were observed in terms of interhemispheric connections, that were particularly evident for Case 1.

In this study Case 1 and Case 2 were compared with a HC template obtained with the same method of Blasi et al. (2020). Although subjects included in the group allowed a good age-match with Case 1 and 2, SC is dependent on age. Therefore, defining an even more homogeneous HC template group is warranted for future studies using SPIDER-NET.

The connectivity patterns of pathological cases with focal lesions were here chosen for test purposes of a novel tool.

Nonetheless, generating SPIDER-NET connectograms could be a good general strategy to test the robustness of the processing pipeline, including the connectivity metrics, further conditioning (e.g., thresholding or binarization), and global or local graph indices. As one of the main limitation of connectomics, so far, is the lack of standardized procedures for network construction and edge weighting (Campbell and Pike, 2014; Maier-Hein et al., 2017), SPIDER-NET may be applied as a flexible and easy tool for calibrating connectomics analyses. Specifically, it could allow to quickly identify expected patterns of disconnection and to easily highlight major errors if present. This quality check may offer a benchmark before addressing less trivial connectivity alterations, as the ones induced by diffused neurodegeneration, which might be another application field of SPIDER-NET.

The last step of graph analysis usually involves the computation of a set of different indices describing network topology and architecture, and dedicated software packages are generally employed. Beyond connectograms generation, SPIDER-NET allows to derive quantitative connectivity metrics, representing global and local (i.e., node level) network properties (Rubinov and Sporns, 2010). For instance, characteristic path length is a global index mirroring communication efficiency within the network, while clustering coefficient is a global measure of network segregation. Among the several graph-based indices, the node degree is a basic local property of a network node, representing the number of connections with other nodes. Characterizing node degree distribution is an important component to identify putative hubs, namely nodes with high node degree, which significantly impact on the network topology. The example of application presented in this study highlighted the impact of graph-based metrics in connectivity analysis. Both local and global metrics derived from the whole-brain networks of Case 1 and Case 2 differed from HC one, as expected (Crofts et al., 2011; Cheng et al., 2019; Li et al., 2021). This quantitative result mirrored the differences qualitatively observed with connectograms. Node degree graphs produced by SPIDER-NET provided an intuitive tool to interactively explore network local properties. A first general visual comparison of Case 1, Case 2, and HCs node degree distribution highlighted that the patients with stroke injury were characterized by lower node degree across the whole brain (Sotelo et al., 2020). Therefore, although the two stroke lesions were limited to a portion of the right hemisphere, an alteration of the whole connectivity pattern was induced (Crofts et al., 2011; Cheng et al., 2019). In addition, it is noteworthy that SPIDER-NET graph-analysis confirmed that subcortical gray matter regions (e.g., the putamen) presented high node degrees in HCs, while these brain areas had lower node degrees in Case 1 and Case 2. This result reflected the prevalent subcortical expression of the two stroke lesions. Also, global graph metrics of segregation and integration were derived with SPIDER-NET, emphasizing the differences between cases with stroke injury and HCs (e.g., a drop in density of 28.9 and 25.2%, respectively, vs. HCs). Reduced connectivity in Case 1 and Case 2 compared to HCs was also numerically paralleled by large differences in all the parameters describing network topology and architecture. Specifically, patients with stroke injury were characterized by lower network integration, segregation, and efficiency. It is remarkable a greater difference in the characteristic path length (–92.4%, –56%) rather than in the clustering coefficient (10.5%, 21.1%) between the HCs and the two cases. The strong effect of the stroke lesion seems to lead to a much more reduced integration than segregation in the contralesional emisphere as shown in Crofts et al. (2011). Furthermore, Case 1 presented larger differences with HCs than Case 2, mirroring the greater clinical severity of the former. Therefore, SPIDER-NET automatically and easily provided useful metrics to quantitatively describe the impairment of the stroke patients included in this study.

Currently, the major limitation of SPIDER-NET is that it allows the analysis of one connectivity matrix at a time. The upload of more than one matrix to extract sub-matrices based on the user selection will be implemented in future SPIDER-NET versions. Furthermore, pre-conditioning operations are currently limited to density thresholding, as this is the most widespread thresholding method in connectivity studies (Wang et al., 2009; van Wijk et al., 2010; Beare et al., 2017). However, at present, other customed approaches can be used prior to the employment of SPIDER-NET by directly uploading already processed matrices. SPIDER-NET offers a flexible sub-network extraction method which relies on *a priori* hypothesis testing by manual selection of parcels/group-parcels and attributes. However, different approaches exist to automatically identify sub-graphs of interest (Hopcroft and Tarjan, 1973; Zalesky et al., 2010), especially in cases in which gross brain abnormalities may not be present. An interesting perspective may be to include automatic and data-driven algorithms for sub-network extraction and comparison with hypothesis-driven selection. In future works, investigating neurological diseases other than stroke and assessing changes associated with treatments (e.g., drugs or rehabilitation) is warranted to test SPIDER-NET sensitivity in detecting brain connectivity changes. Another interesting application might be the investigation of FC with SPIDER-NET and the integration of structural and functional information thanks to the flexibility in extracting sub-networks. In addition, SPIDER-NET application to brain connectivity matrices derived with other modalities (e.g., EEG, MEG, NIRS) could be a further future development. Although SPIDER-NET was presented and tested in this study for MRI datasets, its broad flexibility would actually allow applications even in several other diverse contexts, including all -omics disciplines. For instance, in the framework of rehabilomics (Wagner and Sowa, 2014), which integrates evaluation of transdisciplinary biomarkers, SPIDER-NET may help in the definition of patient-tailored rehabilitative treatments.

## Conclusion

In this work, we proposed a new freely available software package called SPIDER-NET^7^ and we tested it for deriving qualitative and quantitative valuable information of brain connectivity. First, the tool provided a facilitated, interactive, and real-time visualization of connectograms, based on flexible investigation of brain sub-networks. In addition, the automatic computation of topological properties of the networks completed the assessment with quantitative metrics. In conclusion, SPIDER-NET proved to be an accessible and useful tool for human brain connectome investigation in both physiological and pathological conditions.

## Data Availability Statement

The data presented in this study are available on request from the corresponding author. The MRI data are not publicly available due to privacy concerns.

## Ethics Statement

The studies involving human participants were reviewed and approved by IRCCS Fondazione Don Carlo Gnocchi Ethics Committee. The patients/participants provided their written informed consent to participate in this study.

## Author Contributions

DC, GB, and FB contributed to the conception and design of the study. DC developed SPIDER-NET, performed the associated analyses, and wrote the first draft of the manuscript. AP, LP, MC, and ML performed the image processing to derive connectivity matrices. DC, AP, and LP drafted the first version of the manuscript. All authors contributed to manuscript revision, read, and approved the submitted version.

## Conflict of Interest

The authors declare that the research was conducted in the absence of any commercial or financial relationships that could be construed as a potential conflict of interest.

## Publisher’s Note

All claims expressed in this article are solely those of the authors and do not necessarily represent those of their affiliated organizations, or those of the publisher, the editors and the reviewers. Any product that may be evaluated in this article, or claim that may be made by its manufacturer, is not guaranteed or endorsed by the publisher.

## Footnotes

1
https://github.com/paul-kassebaum-mathworks/circularGraph

2
https://sites.google.com/site/bctnet/

3
www.natbrainlab.co.uk

4
http://trackvis.org/

5
http://www.xinapse.com/

6
http://circos.ca/

7
https://caditer.dongnocchi.it
