# Supplementary material for: Development and Testing of SPIDER-NET: An Interactive Tool for Brain Connectogram Visualization, Sub-Network Exploration and Graph Metrics Quantification
Source: Front Neurosci. 2022 Mar 17;16:818385. doi: 10.3389/fnins.2022.818385 (PMC8968144; doi:10.3389/fnins.2022.818385)
Supplement: Supplementary file 1 [file Data_Sheet_1.ZIP › Supplementary Materials/SupplementaryFigure.pdf]

Supplementary materials

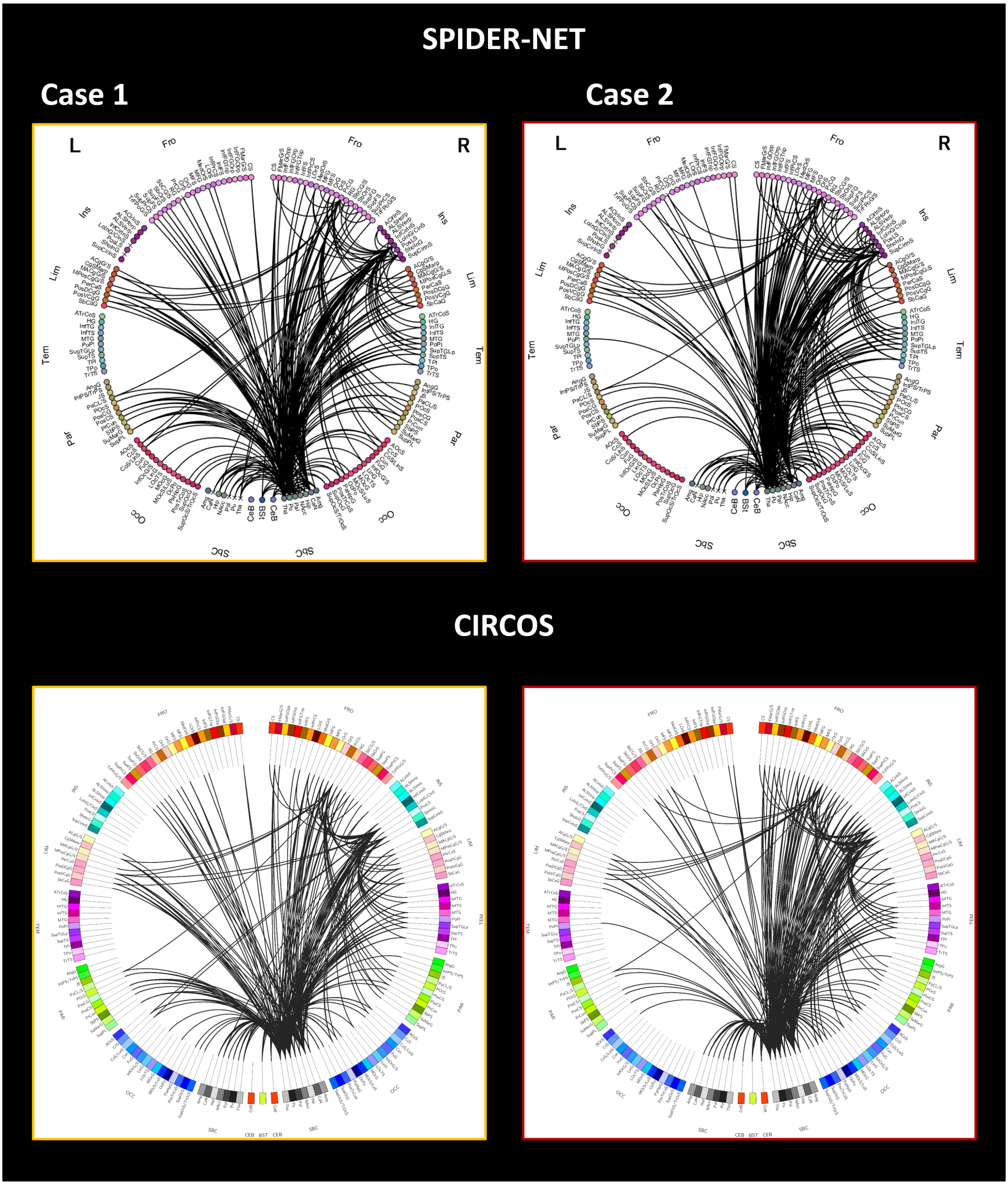

Supplementary Figure 1. Sub-network connectograms generated with SPIDER-NET (upper panel) and with Circos software (http://circos.ca/,) (lower panel). Case 1 connectograms are shown on the left, Case 2 connectograms are shown on the right. The following regions of interest were considered as seeds: right precentral gyrus, right long insular gyrus and central insular sulcus, right short insular gyri, right caudate nucleus, right pallidum, right putamen, and right thalamus.

Legend: L-left hemisphere, R-right hemisphere, Fro-frontal, Ins-insular, Tem-temporal, Par-parietal, Occ-occipital, Sbc-subcortical, CeB-cerebellum, Bst-brainstem. Brain parcels are reported with standard labels provided for the Destrieux atlas.
